# DPAC: A Tool for Differential Poly(A)–Cluster Usage from Poly(A)–Targeted RNAseq Data

**DOI:** 10.1534/g3.119.400273

**Published:** 2019-04-25

**Authors:** Andrew Routh

**Affiliations:** * Department of Biochemistry and Molecular Biology; † Sealy Centre for Structural Biology and Molecular Biophysics, University of Texas Medical Branch, Galveston, Texas, 77555

**Keywords:** Poly(A)-sites, Alternative polyadenylation, Differential gene expression, ClickSeq

## Abstract

Poly(A)-tail targeted RNAseq approaches, such as 3′READS, PAS-Seq and Poly(A)-ClickSeq, are becoming popular alternatives to random-primed RNAseq to focus sequencing reads just to the 3′ ends of polyadenylated RNAs to identify poly(A)-sites and characterize changes in their usage. Additionally, we and others have demonstrated that these approaches perform similarly to other RNAseq strategies for differential gene expression analysis, while saving on the volume of sequencing data required and providing a simpler library synthesis strategy. Here, we present DPAC (***D***ifferential ***P***oly(***A***)-***C***lustering); a streamlined pipeline for the preprocessing of poly(A)-tail targeted RNAseq data, mapping of poly(A)-sites, poly(A)-site clustering and annotation, and determination of differential poly(A)-cluster usage using DESeq2. Changes in poly(A)-cluster usage is simultaneously used to report differential gene expression, differential terminal exon usage and alternative polyadenylation (APA).

The abundance of RNA transcripts as well as poly(A)-site positions can be determined directly from RNAseq techniques that target the junction of 3′UTRs and poly(A) tails. Numerous approaches, including 3′READS (Zheng *et al.* 2016), PAS-Seq (Shepard *et al.* 2011) and Poly(A)-ClickSeq (Routh *et al.* 2017), are commonly and commercially available and can be used to estimate transcript abundance, differential gene expression, alternative terminal exon usage (TE) and alternative polyadenylation (APA). In addition to providing information on the location of poly(A)-sites (PASs) in mRNA transcripts, these methods provide a simple and powerful alternative for gene abundance quantitation to randomly-primed RNAseq in both bulk and single-cell RNAseq experiments. We and others have recently demonstrated that poly(A)-targeted RNAseq approaches perform differential expression analyses similarly to other RNAseq strategies, while saving on the volume of sequencing data required and providing a simpler library synthesis strategy (Elrod *et al.* 2019).

We present DPAC (***D***ifferential ***P***oly(***A***)-***C***lustering) as a pipeline to preprocess raw poly(A)-tail targeted RNAseq data, map to a reference genome, identify and annotate the location of PASs, generate poly(A)-clusters (PACs) and determine the differential abundance of PACs between two conditions. DPAC comprises four major stages; 1) Pre-processing of raw poly(A)-tailed RNAseq including estimation of length of poly(A)-tail tracts; 2) mapping to a reference genome; 3) an optional step that locates all PASs in the provided data and generates annotated poly(A)-clusters (PACs); and 4) a differential expression analysis of PACs using DESeq2. By determining changes in individual PAC abundance, DPAC will calculate changes in terminal exon usage and gene expression by collapsing read counts from individual PACs if they are present on the same exon/intron and whole-gene respectively. DPAC compiles these results and generates a final output table simultaneously describing changes in gene expression, terminal exon (or intron) usage and alternative polyadenylation.

We demonstrate the utility of this pipeline by re-analyzing published 3′READS+ (Zheng *et al.* 2016) and PAS-Seq (Shepard *et al.* 2011) datasets as well as our previously published data using Poly(A)-ClickSeq to measure changes in PAC usage in HeLa cells knocked-down for mammalian Cleavage Factor I 25kDa subunit (CFIm25) (Routh *et al.* 2017). As expected, DPAC reports that CFIm25 depletion results in substantial shortening in 3′UTRs, while only minimally affecting overall gene expression levels. DPAC, along with annotated poly(A)-cluster databases generated in this manuscript, is maintained and available at https://sourceforge.net/projects/DPAC-Seq/

## Materials and Methods

**Figure 1:**
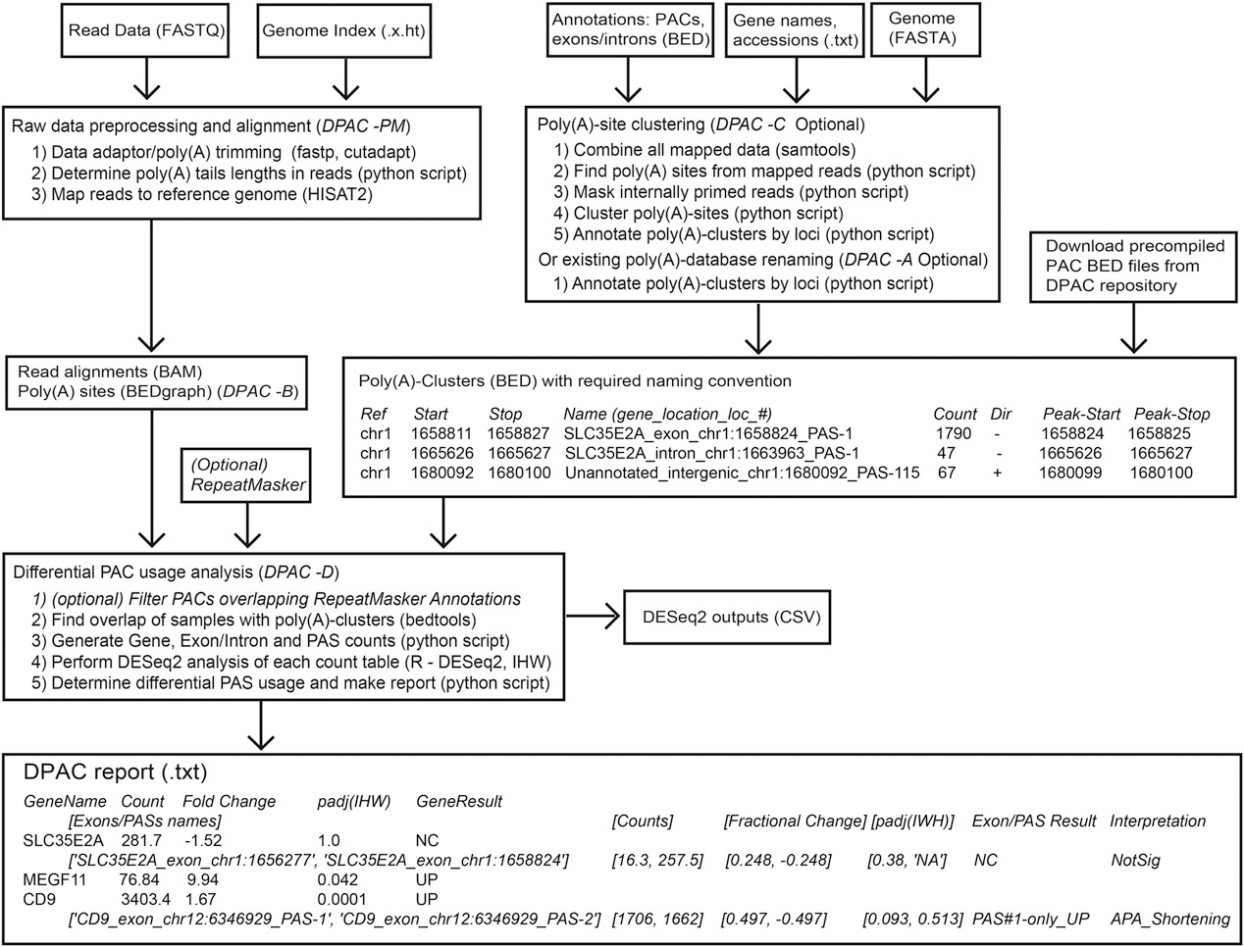
A flow-chart summarizing each of the stages, required input files and returned output files for the DPAC pipeline. Command-line options used to invoke each stage are illustrated: -P for raw data preprocessing, -M for mapping, -C for poly(A) cluster generation, -A for poly(A) cluster database renaming, -B for bedgraphs, -D for the final differential PAC usage analysis. Examples of the output of the DPAC pipeline are shown for three genes: SCL35E2A, MEGF11, and CD9.

DPAC is a simple bash batch script with associated python3 scripts, run with a single command line entry. Details of the locations and identities of raw data are provided by a user-generated tab-delimited metadata file. The pipeline can be broken down into 4 main stages, each of which can be invoked independently to allow re-analysis with new parameters. A number of software dependencies are listed, though these are common in RNAseq pipelines and on bioinformatic servers. A flow chart of each of the main stages of DPAC is shown in Figure 1.

In this manuscript, we differentiate between poly(A)-sites (PASs) and poly(A)-clusters (PACs) as follows: a PAS corresponds to the exact nucleotide of the junction between a 3′UTR and a poly(A) tract to which either one or more sequence reads has mapped; PACs refer specifically to the annotated regions within the genome (whether exonic, intronic or intergenic) in which either a single or multiple clustered PASs are found. The size of the PAC is determined as a function of the DPAC clustering algorithm, which in turn is determined by the chosen clustering window size (described below) and the distance between adjacent PASs.

### Initial data prep and poly(A)-site (PAS) mapping

3′ end sequencing methods including Poly(A)-ClickSeq (PAC-Seq) (Routh *et al.* 2017) generate raw sequence reads overlapping the junction of the 3′ UTR and the poly(A) tail of mRNA transcripts. The preparation of raw read data in terms of adapter trimming, poly(A) tail trimming, poly(A) tail length and quality filtering are essentially the same as previously described (Routh *et al.* 2017). Mapping to a reference genome as well as extraction of poly(A) sites is also performed as previously described. Briefly, reads are trimmed and quality filtered using *fastp* (Chen *et al.* 2018) (parameters: -a AGATCGGAAGAGC -f 6 -g -l 40 –Q). If using approaches such as 3′READS (Zheng *et al.* 2016) where the poly(A)-tail is present in the reverse orientation (*i.e.* a poly(T) tract is present at the beginning of a read), an addition reverse complementation step is performed using the *fastx toolkit* (specified using -c). Trimmed reads are trimmed a second time using *cutadapt* (Martin 2011) to remove and measure the poly(A)-tail returning reads that are longer than a user-defined length (default of 40nts) (parameters: -b A{15} -n 2 -O 10 –m 40). Reads containing poly(A) tracts shorter than 10 A’s are discarded. Next, reads output from this step are compared to the raw data to determine how many A’s (if any) were removed from the 3′ end of the read. This number is appended to the name of each read for future quality filtering. The preprocessing steps of DPAC are invoked by default or by using the ‘–p P’ command-line argument.

**Figure 2:**
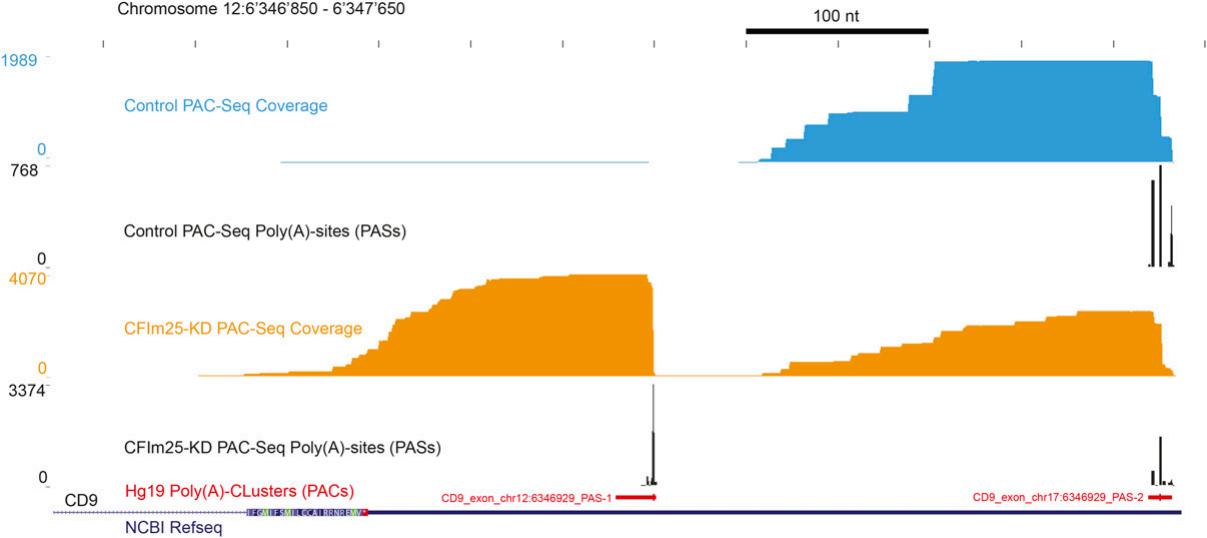
Read coverage and the detected poly(A) sites (PASs) over the CD9 gene for two samples of Poly(A)-ClickSeq analysis of mocked treated HeLa cells (blue) and CFIm25 siRNA treated HeLa cells (orange) are depicted. Poly(A)-Clusters (PACs) are illustrated as a track (red) in the UCSC genome browser. The most frequently detected poly(A)-site within the poly(A)-cluster is highlighted as the thicker portion of the whole poly(A)-cluster in the track.

After data preparation, reads are mapped using default settings to the reference genome using *HISAT2* (Kim *et al.* 2015). The mapping step of DPAC is invoked by default or by using the ‘–p M’ command-line argument. If required, DPAC will also output the individual bedgraphs annotating all poly(A)-sites and mapping coverage for each sample by using the command-line argument ‘–p B’. These files can be loaded into canonical genome browsers and may be useful when generating figures. However, they are not required for the downstream analysis. An example of these output data are shown in Figure 2, illustrating the mapping of PAC-Seq reads and identified PASs for two samples.

### Generating Poly(A)-Clusters (PACs)

The poly(A)-clustering stage of DPAC requires specific BED files of annotated genes, exons and introns which can be obtained from the UCSC genome browser table browser. This is not invoked by default, but by using the command-line entry ‘-p C’. To maximize the power of PAC annotation using PAC-Seq, the data from all samples provided by the user in the metadata file are first combined and the 3′end of all reads are used to identify the location of PASs across the reference genome. The number of A’s found in each poly(A)-tract (as determined in the first pre-processing stage of DPAC) is utilized to score the confidence of each PAS. By default, PASs are output to a raw bedgraph file if a PAS is identified by at least 5 reads each with a poly(A)-tract of at least 25 A’s. These parameters can be adjusted in the command-line and must be chosen carefully depending upon the strategy used for poly(A)-seq library synthesis. In Poly(A)-ClickSeq libraries (Routh *et al.* 2017), the oligo-dT primer used is 21nts but it is not anchored and therefore can prime anywhere within the poly(A) tail. Therefore, by selecting a number greater than 21 (*e.g.*, the default is 25), this ensures that PASs are only reported if a greater number of A’s were in the poly(A)-tail of the sequencing read than can be derived solely from the oligo-dT primer. In the case of PAC-seq, this provides a valuable tool to filter out reads resulting from internal or mis-priming events at the RT stage. For other techniques such as PAS-seq (Shepard *et al.* 2011) and 3′READS+ (Zheng *et al.* 2016), the number of A’s found in the poly(A)-tract at the ends of the read is usually only 10-15nts. In these cases, the number of required A’s in each sequencing read must be reduced accordingly to allow PAS annotation. Finally, PASs are filtered for internal priming by counting the number of A’s in the reference genome immediately downstream of the identified PASs. If 12 or more A’s are found within 20 nts downstream, these events are ‘masked’ and not further utilized.

PASs are predominantly found at a ‘GA’, ‘UA’ or ‘CA’ dinucleotides, although the exact site is variable (Routh *et al.* 2017; Derti *et al.* 2012). By default, single PASs occurring within 25 nts of one another are merged into poly(A)-clusters (PACs), which are subsequently treated as singular features in downstream analyses. To annotate PACs, exon and intron annotations are first obtained from the UCSC database. The overlap of each PAC to annotated exons and introns is then determined using *bedtools* (Quinlan and Hall 2010). PACs are annotated according to: the gene name; whether the PAC is exonic, intronic or found just downstream of a terminal exon; the genomic coordinate; and finally assigned a number depending upon the number of other PACs found within the same exon or intron (for example see Table 1). PACs found in intergenic or otherwise unannotated sequences are numbered sequentially depending upon the total number of unannotated PACs found. This naming scheme is used in the final stage of DPAC to differentiate between alternative polyadenylation events and alternative terminal exon usage.

**Table 1:** Example of count table used or DESeq2 for CD9, CD9 exon, and CD9 poly(A)-clusters

*Table*	*Gene/Exon/PAC*	*Ctrl1*	*Ctrl2*	*Ctrl3*	*CFIm25-Kd1*	*CFIm25-Kd2*	*CFIm25-Kd3*
Gene:	CD9	1993	1820	1900	6639	4021	6806
Exon:	CD9_exon_chr12:6346929	1993	1820	1900	6639	4021	6806
PACs:	CD9_exon_chr12:6346929_PAS-1	5	267	388	4061	2537	4262
	CD9_exon_chr12:6346929_PAS-2	1988	1553	1512	2578	1484	2544

Rather than performing *de novo* PAC annotation, pre-existing databases of poly(A)-clusters generated by DPAC, such as the ones used in this report, can be found online at https://sourceforge.net/projects/dpac-seq/files/Poly(A)_Clusters_BED/. Alternatively, other established poly(A) databases such as from the PolyA_DB (Lee *et al.* 2007; Zhang *et al.* 2005) can be provided to DPAC. As the naming conventions for PACs described above is essential for the downstream stages of DPAC, the ‘-p A’ argument must be selected in these instances to evoke a short script that will rename and sort the PACs.

Examples of two specific poly(A)-clusters within the CD9 gene are illustrated in Figure 2 in the hg19 Poly(A)-Cluster track in red. Information regarding the most frequent PAS nucleotide or each PAC is retained as extra columns in the output BED file, illustrated as the thicker BED line Poly(A)-Cluster track.

### Determination of differential Poly(A)-Cluster usage using DESeq2

In the final stage of DPAC, the mapped reads from each individual samples are used to determine the frequency of PACs in each dataset by determining the overlaps of the 3′ ends of the mapped reads with the provided poly(A) cluster database using *bedtools* (Quinlan and Hall 2010). A PAC is counted if the 3′ end of a mapped read overlaps within a user-defined distance (10nts by default) of an annotated poly(A)-cluster and count tables of PACs are returned. Next, if multiple PACs are found within an exon or intron, then these are collapsed into a single entry, generating a new count table for exons and introns. Similarly, if multiple PACs are found within a single gene, these are also collapsed to create a count table just for whole genes.

By default, only PACs found in exonic regions are collapsed into gene counts as introns can often contain repetitive and/or transposable elements whose inclusion can artificially inflate count numbers. However, intronic PACs can play important roles in the regulation of gene expression, particularly for long transcripts (Wang *et al.* 2019). Therefore, this parameter can be overturned to force inclusion of intronic PASs by selecting ‘**-i**’ in the command-line. In this case, to help prevent gene-count inflation from reads mapping to repetitive elements that may potentially be mis-mapped, an additional option (‘-m’) is provided to filter out PACs that overlap with annotated repetitive and mobile elements (provided by the user with an additional BED or GTF file), such as from the RepeatMasker database (Smit *et al.* 2013–2015).

Three sets of count tables are thus generated (see example counts for CD9 and its exons/PACs in Table 1) and passed individually into DESeq2 (Love *et al.* 2014). Data normalization and statistical tests are applied using the canonical DESeq2 pipeline using *local* dispersion estimation and Independent Hypothesis Weighting (IHW) (Ignatiadis *et al.* 2016) to estimate false discovery rates and for power maximization. Thus, differential usage of PACs, exons/introns and whole-genes are calculated and the results are output as csv files. As illustrated in the flow-chart in Figure 1, these files are returned for inspection, figure generation and other downstream analyses.

### Output

After DESeq2 analysis, a final compiled table is generated containing information about PAC usage for each gene (including only exons unless the **–i** option is selected). If a gene only has one PAC and thus one terminal exon, only the gene information is returned. Genes with differential expression (fold-change > 1.5; *padj* < 0.1) are annotated as ‘DOWN’ or ‘UP’. If there is no significant change, genes are labels as ‘NC’ (No Change). Alternative polyadenylation (APA) or differential terminal exon usage (TE) is reported when a gene has two or more PACs (minimum occupancy of 5% per PAC), with at least one PAC undergoing differential usage with an IHW *padj* <0.1 and resulting in a fractional change of the PAC usage by at least 10%. If two PACs are found in different exons, this is annotated as a differential terminal exon usage event (denoted as ‘TE’). If the two PACs are found in the same exon, then this annotated as an APA event. The relative locations of the PACs is then used to determine whether the APA results in 3′UTR shortening or lengthening. If three or more PACs (again with minimum occupancy of 5% per PAC) are found within a single exon and one of the middle PACs changes in abundance, then this can simultaneously result in changes of abundance of both upstream and downstream PACs, resulting in both a shortening and a lengthening phenotype. These are annotated as ‘APA_both’.

### Data Availability Statement

All data used in the manuscript is available at the NCBI SRA database as deposited by their respective authors: HeLa cell PAC-Seq data (PRJNA374982); HeLa cell 3′READs+ data (PRJNA328218); and MEF cell PAS-seq data (PRJNA436720). Annotated PAC datasets generated in this manuscript are available in supplementary material and at https://sourceforge.net/projects/dpac-seq/files/Poly%28A%29_Clusters_BED/ DPAC is freely available (MIT license), is maintained and available at https://sourceforge.net/projects/DPAC-Seq. Supplemental material available at FigShare: https://doi.org/10.25387/g3.7635971.

## Results

### Re-analysis of Poly(A)-ClickSeq data of CFIm25 knock-down in HeLa cells

To evaluate this pipeline, we re-analyzed the PAC-seq data deposited at NCBI SRA (*PRJNA374982*) from the original PAC-Seq publication (Routh *et al.* 2017). The six datasets were derived from total cellular RNA extracted from three technical replicates each of mock-treated and CFIm25 KD HeLa cells. We applied our pipeline to locate and annotate *de novo* poly(A)-clusters (PACs) and then to determine the differential usage of PACs between each condition. Annotation data and example command-line entries are provided in the DPAC manual to repeat these analyses.

During *de novo* PAS clustering, a total of 44,422 poly(A) clusters (PACs) with >25 reads were identified in the datasets. Of these, 27,958 were exonic, 7,441 were intronic, 928 were found within 250nts downstream of annotated 3′ terminal exons; and 8,094 were intergenic or otherwise unannotated (**Supplementary Data 1**).

To detect differential poly(A)-cluster usage, we performed the final stage of DPAC using the three following conditions: 1) only considering exonic PACs, 2) considering all PACs (exonic or otherwise) but filtering out PACs overlapping with the hg19 RepeatMasker database; and 3) considering all PACs (exonic or otherwise) but using the poly(A) database (PolyA_DB) instead of *de novo* PAC annotation. Summaries of the output are shown in Table 2. Reports of both differential gene expression and differential PACs usage (IHW-padj < 0.1, fold-change >1.5) are provided in **Supplemental Datasets 2, 3** and **4**.

**Table 2:** Summaries of findings of DPAC analysis of CFIm25 KD HeLa cells using three different sets of parameters

	Exons only	All PACs (inc. introns)	PolyA_DB PACs
**Genes mapped**	12499	12886	11523
- Increase	267	335	261
- Decrease	117	121	103
**Exons or introns mapped**	14025	26217	14367
- Increase	342	412	392
- Decrease	130	146	127
Terminal Exon Change	154	235	194
**PACs mapped**	29411	41573	20949
- Increase	1167	925	1052
- Decrease	307	308	271
Genes with multiple PACs	5067	5880	3881
**Genes undergoing APA**	861	647	638
- Shortening	620	457	485
- Lengthening	82	78	89
- Both	153	109	60

**Figure 3:**
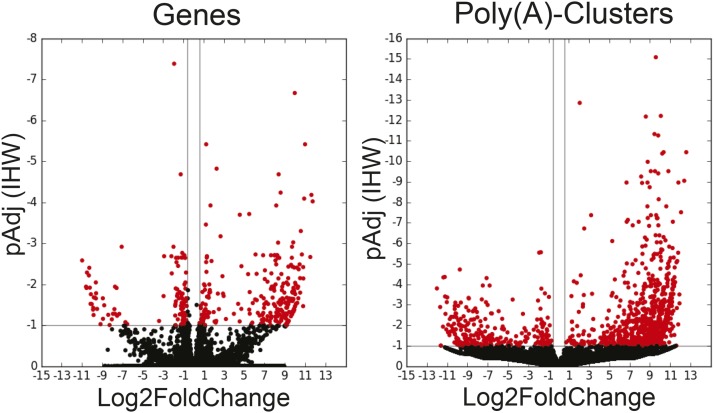
Volcano plots of the differential expression of Genes (left) and Poly(A)-Clusters (right) in HeLa cells upon siRNA KD of CFIm25 using default settings of DPAC (data from Table 2, column 1). Red dots indicate genes or PACs with a fold changes greater than 1.5 and a p-adjusted value less than 0.1.

When considering all PACs (including intronic) our pipeline found PACs mapped over a total of 12,886 genes, of which 5,880 (47%) exhibited multiple PACs (Table 2), similar to rates previously reported (Routh *et al.* 2017; Lee *et al.* 2007). By virtue of measuring differential usage of each individual PAC independently, DPAC revealed differential usage of PACs outside of annotated regions. Indeed, of the total 1,233 identified differentially expressed PACs, 94 (7.6%) were intronic and 99 (8.0%) were found in unannotated regions. Volcano plots illustrating changes in gene expression and PAC abundance are shown in Figure 3. Due to changes in PAC abundance, DPAC reported that 647 genes exhibited APA with the shortening of 457 3′UTRs and lengthening of 78 3′UTRs. 109 exons exhibited both lengthening and shortening, due to the presence of multiple PACs within 3′UTRs. The predominant shortening of 3′UTRs upon knock-down of CFIm25 is the expected phenotype and is consistent with our and others' previous analyses (Routh *et al.* 2017; Zhu *et al.* 2018; Chu *et al.* 2019). Differential PAC usage resulted in alternative terminal exon usage in 236 genes and only 25 genes exhibited APA and TE simultaneously.

DPAC outputs a report detailing changes in gene expression, exon usage and PAC usage (**Supplemental Datasets 2, 3** and **4).** Specific examples of the final output are shown in Figure 1. SLC35E21 has two PACs found in two different terminal exons, but there is no significant change in their usage and therefore no alternative terminal exon usage. MEGF11 has only one PAC, and this gene is significantly up-regulated upon CFIm25 KD. CD9 is also up-regulated upon CFIm25 KD due to the up-regulation of one of two PACs found within the same terminal exon with a net effect of 3′UTR shortening. The mapping of the raw data over CD9 and the detected PASs and PACs are shown in Figure 2.

### Re-analysis of 3′READs+ and PAS-Seq data

The DPAC pipeline was conceived during the development of analysis of Poly(A)-ClickSeq datatypes. Nonetheless, the DPAC pipeline is applicable to any data type provided that there are poly(A) tracts (or poly(T) tracts in the negative sense) retained within the read data that are of at least 10nts in length and the read length after poly(A) trimming is greater than 25nts. There are many current poly(A)-tail focused methods for RNAseq (Zhang *et al.* 2018), that yield similar read data focused on the 3′UTR and poly(A)-tail junction. So to demonstrate this functionality, we ran the DPAC pipeline using previously deposited and published datasets to generate *de novo* PAC datasets: 1) 3′READs+ data derived from HeLa cells (human) (Zheng *et al.* 2016) and 2) PAS-Seq datasets derived from MEF cells (murine) (Chang *et al.* 2018). Summaries of the output are shown in Table 3 and the final PAC datasets (BED format) are available in **Supplementary Datafiles 5** and **6** respectively.

**Table 3:** Summaries of Poly(A)-Clusters annotated using 3′READs+ and PAS-Seq datasets

*Strategy*	*Article*	*Genome*	*Raw Reads*	*Processed Reads*	*PACs discovered*	*Parameters Used (other than default)*
**3′READs+**50SE HiSeq	(Zheng *et al.* 2016)	hg19 *Homo sapiens*	29,820,497 4 datasets	2,519,867	21,532 Total, 15,599 Exonic, 2,081 Intronic, 3,852 Intergenic	‘-c’ (*Reverse Complement*), ‘-a 10’ (*Minimum A-tract length*), ‘-l 25’ (*Minimum read length*)
**PAS-Seq** 100SE HiSeq	(Chang *et al.* 2018)	mm10 *Mus musculus*	83,224,679 4 datasets	37,663,516	34,156 Total, 19,922 Exonic, 4,834 Intronic, 9,402 Intergenic	‘-a 10’ (*Minimum A-tract length*)

## Discussion

In summary, DPAC performs each of the necessary steps required for preprocessing, poly(A)-site identification, poly(A)-clustering and differential PAC usage required for poly(A)-targeted RNAseq experiments. Our pipeline is suitable for analysis of multiple different strategies for poly(A)-tail sequencing, provided that stretches of the poly(A)-tail greater than 10nts are retained within the sequencing data. We further recommend that read-lengths are sufficiently long, once accounting for removal of the poly(A)-tract, so that at least 40nts of 3′UTR sequence remain. While shorter reads can be tolerated (*e.g.*, as demonstrated in Table 3), this ensures that reads are mapped unambiguously, which may be particularly important for large genomes.

In principle, DPAC may also be used to analyze canonical random-primed RNAseq data or RNAseq data enriched coarsely for 3′UTRs, such as in QuantSeq (Moll *et al.* 2014), as many of these reads will map over the junction of the 3′UTR and poly(A) by chance, although the frequency of these reads may be low. However, there exist other sophisticated tools for poly(A)-site annotation and measurement of alternative polyadenylation such as DaPars (Xia *et al.* 2014) and TAPAS (Arefeen *et al.* 2018) that are designed specifically for these data types.

DPAC reports the expected findings upon reanalysis of Poly(A)-ClickSeq datasets comparing mock and CFIm25-knockdown HeLa cells. By virtue of assessing changes in all PACs regardless of whether they are found in annotated genomic regions, DPAC may also allow discovery of novel mRNA transcripts and/or changes in the expression of ncRNAs and/or non-coding transposable elements. DPAC therefore provides a singular pipeline to simultaneously report differential gene expression, terminal exon usage and alternative polyadenylation.

## References

[bib1] Arefeen A. Liu J. Xiao X. Jiang T., 2018 TAPAS: tool for alternative polyadenylation site analysis. Bioinformatics 34: 2521–2529. 10.1093/bioinformatics/bty110 30052912 PMC6454472

[bib2] Chang J. W. Zhang W. Yeh H. S. Park M. Yao C., 2018 An integrative model for alternative polyadenylation, IntMAP, delineates mTOR-modulated endoplasmic reticulum stress response. Nucleic Acids Res. 46: 5996–6008. 10.1093/nar/gky340 29733382 PMC6158760

[bib3] Chen S. Zhou Y. Chen Y. Gu J., 2018 fastp: an ultra-fast all-in-one FASTQ preprocessor. Bioinformatics 34: i884–i890. 10.1093/bioinformatics/bty560 30423086 PMC6129281

[bib4] Chu Y. Elrod N. Wang C. Li L. Chen T., 2019 Nudt21 regulates the alternative polyadenylation of Pak1 and is predictive in the prognosis of glioblastoma patients. Oncogene. 10.1038/s41388-019-0714-9 PMC6533131 30705404

[bib5] Derti A. Garrett-Engele P. Macisaac K. D. Stevens R. C. Sriram S., 2012 A quantitative atlas of polyadenylation in five mammals. Genome Res. 22: 1173–1183. 10.1101/gr.132563.111 22454233 PMC3371698

[bib6] Elrod N. R. Jaworski E. A. Ji P. Wagner E. J. Routh A., 2019 Development of Poly(A)-ClickSeq as a Tool Enabling Simultaneous Genome-wide Poly(A)-site identification and Differential Expression Analysis. Methods 155: 20–29. 10.1016/j.ymeth.2019.01.002 30625385 PMC7291597

[bib7] Ignatiadis N. Klaus B. Zaugg J. B. Huber W., 2016 Data-driven hypothesis weighting increases detection power in genome-scale multiple testing. Nat. Methods 13: 577–580. 10.1038/nmeth.3885 27240256 PMC4930141

[bib8] Kim D. Langmead B. Salzberg S. L., 2015 HISAT: a fast spliced aligner with low memory requirements. Nat. Methods 12: 357–360. 10.1038/nmeth.3317 25751142 PMC4655817

[bib9] Lee J. Y. Yeh I. Park J. Y. Tian B., 2007 PolyA_DB 2: mRNA polyadenylation sites in vertebrate genes. Nucleic Acids Res. 35: D165–D168. 10.1093/nar/gkl870 17202160 PMC1899096

[bib10] Love M. I. Huber W. Anders S., 2014 Moderated estimation of fold change and dispersion for RNA-seq data with DESeq2. Genome Biol. 15: 550 10.1186/s13059-014-0550-8 25516281 PMC4302049

[bib11] Martin M., 2011 Cutadapt removes adapter sequences from high-throughput sequencing reads. *EMBnet.journal* 17 (1):10–12.

[bib12] Moll P. Ante M. Seitz A. Reda T., 2014 QuantSeq 3′ mRNA sequencing for RNA quantification. Nat. Methods 11: i–iii. 10.1038/nmeth.f.376

[bib13] Quinlan A. R. Hall I. M., 2010 BEDTools: a flexible suite of utilities for comparing genomic features. Bioinformatics 26: 841–842. 10.1093/bioinformatics/btq033 20110278 PMC2832824

[bib14] Routh A. Ji P. Jaworski E. Xia Z. Li W., 2017 Poly(A)-ClickSeq: click-chemistry for next-generation 3-end sequencing without RNA enrichment or fragmentation. Nucleic Acids Res. 45: e112 10.1093/nar/gkx286 28449108 PMC5499544

[bib15] Shepard P. J. Choi E. A. Lu J. Flanagan L. A. Hertel K. J., 2011 Complex and dynamic landscape of RNA polyadenylation revealed by PAS-Seq. RNA 17: 761–772. 10.1261/rna.2581711 21343387 PMC3062186

[bib16] Smit, A., R. Hubley, and P. Green, 2013–2015 RepeatMasker Open-4.0. http://www.repeatmasker.org.

[bib17] Wang R. Zheng D. Wei L. Ding Q. Tian B., 2019 Regulation of Intronic Polyadenylation by PCF11 Impacts mRNA Expression of Long Genes. *Cell Rep* 26 (10):2766–2778 e2766. 10.1016/j.celrep.2019.02.049 PMC6428223 30840896

[bib18] Xia Z. Donehower L. A. Cooper T. A. Neilson J. R. Wheeler D. A., 2014 Dynamic analyses of alternative polyadenylation from RNA-seq reveal a 3′-UTR landscape across seven tumour types. Nat. Commun. 5: 5274 10.1038/ncomms6274 25409906 PMC4467577

[bib19] Zhang H. Hu J. Recce M. Tian B., 2005 PolyA_DB: a database for mammalian mRNA polyadenylation. Nucleic Acids Res. 33: D116–D120. 10.1093/nar/gki055 15608159 PMC540009

[bib20] Zhang Y. Carrion S. A. Zhang Y. Zhang X. Zinski A. L., 2018 Alternative polyadenylation analysis in animals and plants: newly developed strategies for profiling, processing and validation. Int. J. Biol. Sci. 14: 1709–1714. 10.7150/ijbs.27168 30416385 PMC6216028

[bib21] Zheng D. Liu X. Tian B., 2016 3′READS+, a sensitive and accurate method for 3′ end sequencing of polyadenylated RNA. RNA 22: 1631–1639. 10.1261/rna.057075.116 27512124 PMC5029459

[bib22] Zhu, Y., X. Wang, E. Forouzmand, J. Jeong, F. Qiao *et al.*, 2018 Molecular Mechanisms for CFIm-Mediated Regulation of mRNA Alternative Polyadenylation. *Mol Cell* 69 (1):62–74 e64. 10.1016/j.molcel.2017.11.031 PMC5756121 29276085

